# Ki-67 as prognostic marker in early breast cancer: a meta-analysis of published studies involving 12 155 patients

**DOI:** 10.1038/sj.bjc.6603756

**Published:** 2007-04-24

**Authors:** E de Azambuja, F Cardoso, G de Castro, M Colozza, M S Mano, V Durbecq, C Sotiriou, D Larsimont, M J Piccart-Gebhart, M Paesmans

**Affiliations:** 1 Medical Oncology Clinic, Jules Bordet Institute, 125 Boulevard de Waterloo, 1000, Brussels, Belgium; 2 PhD student in the Programa de Pós-graduação em Medicina, Ciências Médicas, Faculdade de Medicina, Universidade Federal do Rio Grande do Sul, 2400 Ramiro Barcelos, 90035-003, Porto Alegre, Brazil; 3 SC Oncologia Medica, Azienda Ospedaliera, Via Brunamonti, 51-06122, Perugia, Italy; 4 Data Centre, Jules Bordet Institute, 125 Boulevard de Waterloo, 1000, Brussels, Belgium

**Keywords:** breast cancer, Ki-67, prognostic value, meta-analysis

## Abstract

The Ki-67 antigen is used to evaluate the proliferative activity of breast cancer (BC); however, Ki-67's role as a prognostic marker in BC is still undefined. In order to better define the prognostic value of Ki-67/MIB-1, we performed a meta-analysis of studies that evaluated the impact of Ki-67/MIB-1 on disease-free survival (DFS) and/or on overall survival (OS) in early BC. Sixty-eight studies were identified and 46 studies including 12 155 patients were evaluable for our meta-analysis; 38 studies were evaluable for the aggregation of results for DFS, and 35 studies for OS. Patients were considered to present positive tumours for the expression of Ki-67/MIB-1 according to the cut-off points defined by the authors. Ki-67/MIB-1 positivity is associated with higher probability of relapse in all patients (HR=1.93 (95% confidence interval (CI): 1.74–2.14); *P*<0.001), in node-negative patients (HR=2.31 (95% CI: 1.83–2.92); *P*<0.001) and in node-positive patients (HR=1.59 (95% CI: 1.35–1.87); *P*<0.001). Furthermore, Ki-67/MIB-1 positivity is associated with worse survival in all patients (HR=1.95 (95% CI: 1.70–2.24; *P*<0.001)), node-negative patients (HR=2.54 (95% CI: 1.65–3.91); *P*<0.001) and node-positive patients (HR=2.33 (95% CI: 1.83–2.95); *P*<0.001). Our meta-analysis suggests that Ki-67/MIB-1 positivity confers a higher risk of relapse and a worse survival in patients with early BC.

The crude incidence of breast cancer (BC) in Europe is 109.8/100.000 women per year and it is responsible for 38.4 out of 100.000 deaths per women annually (Pestalozzi *et al*, 2005). Significant improvements in both disease-free survival (DFS) and overall survival (OS) have been obtained with the extensive use of adjuvant systemic therapies (EBCTCG, 2005). In the last few decades, proliferation markers have been extensively evaluated as prognostic tools in BC. However, the only prognostic factors utilised in clinical decision making are some histologic features (e.g. tumour size, histologic grade, nodal status and lymphovascular invasion), hormone receptor status, HER-2 status and age (Colozza *et al*, 2005; Hayes, 2005).

Ki-67 is present in all proliferating cells and there is great interest in its role as a marker of proliferation (Gerdes *et al*, 1983). The Ki-67 antibody reacts with a nuclear non-histone protein of 395 KD present in all active phases of the cell cycle except the G0 phase (Cattoretti *et al*, 1992). MIB-1 is a monoclonal antibody against recombinant parts of the Ki-67 antigen; a good correlation exists between Ki-67 and MIB-1 (Cattoretti *et al*, 1992).

Recently, gene array techniques have revealed the Ki-67 gene's role in several ‘proliferation signatures’, showing that a set of genes with increased expression patterns is correlated with tumour cell proliferation rates, as assessed by the Ki-67 labelling index (Perou *et al*, 1999; Whitfield *et al*, 2006). Moreover, Ki-67 is one of the 21 prospectively selected genes of the Oncotype DXTM assay used to predict the risk of recurrence in a node-negative, tamoxifen-treated BC population enrolled in the National Surgical Adjuvant Breast and Bowel Project B-14 (NSABP B-14), as well to predict the magnitude of chemotherapy benefit in women with node-negative, estrogen receptor (ER)-positive BC enrolled in the NSABP B20 trial (Paik *et al*, 2004, 2006).

Despite the large number of published papers analyzing the prognostic role of Ki-67 in early BC, it is still not considered as an established factor to be used in clinical practice, probably because most of the studies are retrospective and because some uncertainty remains on the way Ki-67 should be assessed (Eifel *et al*, 2001; Goldhirsch *et al*, 2003; Colozza *et al*, 2005; Urruticoechea *et al*, 2005). Therefore, due to the fact that a more convincing demonstration of the Ki-67 prognostic role, in early BC, would be of value for initiating further research on the assessment methods of Ki-67, we performed this literature-based meta-analysis to better quantify the prognostic impact of Ki-67 expression.

## MATERIALS AND METHODS

### Publication selection

For this meta-analysis, we selected studies evaluating the relationship between Ki-67/MIB-1 status and prognosis in early BC published until May 2006. To fulfill our selection criteria, the studies had to have been published as a full paper in English. Articles were identified by an electronic PubMed search using the following keywords: ‘breast cancer’,‘Ki-67’,‘MIB-1’,‘proliferative index’, ‘proliferative marker’, ‘survival’ and ‘prognostic’. We also screened references from the relevant literature, including all the identified studies and reviews. To avoid duplicate data, we identified articles that included the same cohort of patients by reviewing interstudy similarity in the country in which the study was performed, investigators in the study, source of patients, recruitment period and inclusion criteria. Therefore, when the authors reported the same patient population in several publications, only the most recent or complete study was included in this analysis.

### Data extraction

Information was carefully extracted from all publications by three authors (EA, GC and MP). The following data were collected from each study: publication date, first author's last name, antibody and cut-off used for assessing Ki-67 positivity, distribution of Ki-67 status, follow-up period, treatment, nodal status and data allowing us to estimate the impact of Ki-67 expression on DFS and/or OS.

We did not define any minimal number of patients to include a study in our meta-analysis, nor a minimal duration of median follow-up. The exclusion criteria are described below and were not driven by the study individual results.

### Statistical methods

Ki-67 was considered positive or negative according to the cut-off values provided by the authors. For the quantitative aggregation of the survival results, the impact of Ki-67 expression on prognosis was measured using Hazard Ratio (HR). For each study, this HR was estimated by a method depending on the results provided in the original publication. The most accurate method was to retrieve the estimated HR and its variance using two of the following parameters: the HR point estimate, the log-rank statistic or its *P*-value, and the O–E statistic (difference between numbers of observed and expected events) or its variance. If those data were not available, we looked for the total number of events, the number of patients at risk in each group and the log-rank statistic or its *P*-value, to estimate the HR. Finally, if the only useful data were in the form of graphical representations of the survival distributions, we extracted from them the survival rates at specified time-points in order to reconstruct the HR estimate and its variance, with the assumption that the rate of patients censored was constant during the study follow-up (Parmar *et al*, 1998).

Three independent persons read the curves to reduce reading variability. If authors reported survival of three or more groups, we pooled the results to make feasible a comparison between two groups. Whenever possible, HR estimates for subgroups were calculated, such as in node-negative, node-positive or untreated patients. Results were crosschecked with those from the original publication to be sure that they are not discrepant, in particular when reading of the survival rates had to be performed on the survival curves.

The individual HR estimates were combined into an overall HR using the Peto's method that was first used and published in 1985 (Yusuf *et al*, 1985). We carried out heterogeneity *χ*^2^-tests, and if the assumption of homogeneity of individual HRs had to be rejected, we used a random-effect model in place of a fixed-effect model. By convention, an observed HR>1 implied a worse prognosis for the group with positive Ki-67 expression. This impact of Ki-67 on survival was considered to be statistically significant if the 95% confidence interval (CI) for the overall HR did not overlap 1. We have used the authors’ definitions for DFS and OS.

All the statistical calculations for our meta-analysis were performed with personal computing.

## RESULTS

### Characteristics of the studies

Out of 68 studies published between the years 1989 and 2006, 46 had the sufficient information for HR extraction, including 38 studies evaluable for DFS and 35 for OS, some of them being evaluable for only one of these end points, or they analysed only one of these end points. Tables 1 and 2 list the evaluable studies with their main characteristics, and Table 3 presents the main results of this meta-analysis. The reasons to consider an article as non-evaluable were: (a) no univariate analysis reported; (b) no possibility to calculate HR using one of the methods mentioned above due to the fact that the distribution of Ki-67 was not reported in the article, or sometimes Ki-67 was analysed in combination with other prognostic markers rendering the analysis impossible; (c) overlapping data published in different journals; and (d) inclusion of metastatic BC patients. Table 4 lists all the studies considered non-evaluable for this meta-analysis, but used at sensitivity analysis.

The number of patients included across all studies varied from 42 to 863, and the follow-up period varied from 23.6 months (mean) to 16.3 years (median). Different antibodies were used through all trials: anti-Ki-67 was used in 24 studies (52.1%), anti-MIB-1 in 24 studies (52.1%), both antibodies were performed in five studies (Keshgegian and Cnaan, 1995; Veronese *et al*, 1995; Bevilacqua *et al*, 1996; Querzoli *et al*, 1996; Billgren *et al*, 2002), anti-Ki-S5 in two studies (Rudolph *et al*, 1999a; Esteva *et al*, 2004) and anti-Ki-S11 in one study (Rudolph *et al*, 1999b). The different cut-off values used were those of the authors (range: 3.5–34%). Threshold definitions were mean or median values, the best cut-off value or an established arbitrary value.

Out of the 38 evaluable studies for DFS (10 954 patients), subgroup analysis was possible in 15 studies with node-negative patients (3370 patients) (Sahin *et al*, 1991; Weikel *et al*, 1991, 1995; Gaglia *et al*, 1993; Bevilacqua *et al*, 1996; Brown *et al*, 1996; Pierga *et al*, 1996; Railo *et al*, 1997; Jansen *et al*, 1998; Clahsen *et al*, 1999; Harbeck *et al*, 1999; Rudolph *et al*, 1999a; Billgren *et al*, 2002; Trihia *et al*, 2003; Erdem *et al*, 2005), in eight with node-positive patients (1430 patients) (Weikel *et al*, 1991, 1995; Gaglia *et al*, 1993; Pierga *et al*, 1996; Jansen *et al*, 1998; Billgren *et al*, 2002; Trihia *et al*, 2003; Esteva *et al*, 2004) and in six with untreated node-negative patients (736 patients) (Sahin *et al*, 1991; Weikel *et al*, 1991; Bevilacqua *et al*, 1996; Railo *et al*, 1997; Jansen *et al*, 1998; Harbeck *et al*, 1999). Regarding OS (9472 patients), of all 35 studies, subgroup analysis was possible in nine studies with node-negative patients (1996 patients) (Jensen *et al*, 1995; Weikel *et al*, 1995; Bevilacqua *et al*, 1996; Brown *et al*, 1996; Domagala *et al*, 1996; Fresno *et al*, 1997; Rudolph *et al*, 1999a; Trihia *et al*, 2003; Erdem *et al*, 2005), in four with node-positive patients (857 patients) (Weikel *et al*, 1995; Domagala *et al*, 1996; Gonzalez *et al*, 2003; Trihia *et al*, 2003) and in two that included only untreated patients (node-negative and node-positive) (284 patients) (Pinder *et al*, 1995; Bevilacqua *et al*, 1996).

### Meta-analysis

**Figure 1:**
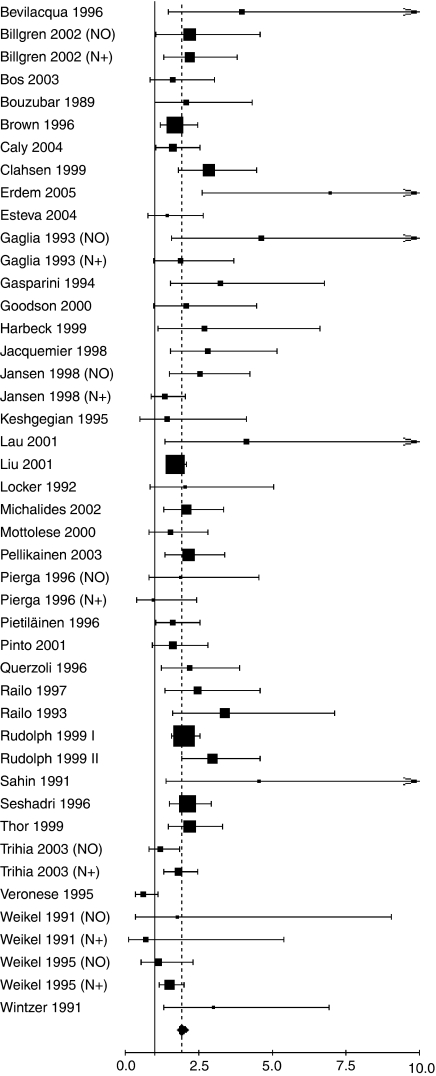
Results of the meta-analysis with all evaluable studies for DFS. A hazard ratio (HR)>1 implies a worse DFS for the group with increased Ki-67. The squared size is proportional to the number of patients included in each study. The centre of the lozenge gives the combined HR for the meta-analysis and its extremities the 95% CI.

**Figure 2:**
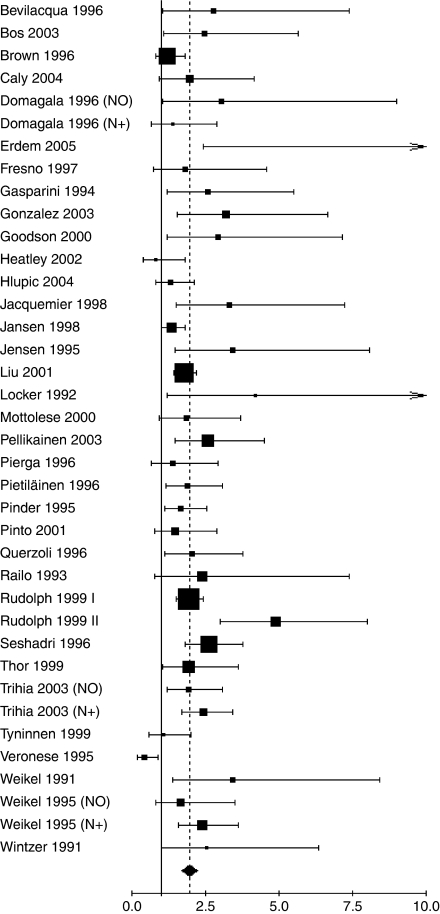
Results of the meta-analysis with all evaluable studies for OS. A HR>1 implies a worse OS for the group with increased Ki-67. The squared size is proportional to the number of patients included in each study. The centre of the lozenge gives the combined HR for the meta-analysis and its extremities the 95% CI.

The main meta-analyses results (overall population and DFS/OS) are shown in Figures 1 and 2. For the overall population, worse DFS (HR 1.93, 95% CI 1.74–2.14; *P*<0.001) and OS (HR 1.95, 95% CI 1.70–2.24; *P*<0.001) were observed among patients considered as Ki-67 positive. Worse prognosis was observed independently both in node-negative (DFS (HR 2.31, 95% CI 1.83–2.92; *P*<0.001); OS (HR 2.54, 95% CI 1.65–3.91; *P*<0.001)) and in node-positive patients (DFS (HR 1.59, 95% CI 1.35–1.87; *P*<0.001); OS (HR 2.33, 95% CI 1.83–2.95; *P*<0.001)). For the untreated patients subgroup analysis, worse DFS was found in all node-negative patients (HR 2.72, 95% CI 1.97–3.75; *P*<0.001), as well as worse OS in node-negative and node-positive patients taken together (HR1.79, 95% CI 1.22–2.63; *P*=0.001).

The necessity to exclude some studies due to a lack of results for aggregating the results is a well-known important problem when conducting a meta-analysis, because the excluded studies show often a smaller effect compared to the studies published with full details and evaluable for the meta-analysis. To assess the impact of bias related to the unevaluable studies (that might lead to an overestimation of the effect), we performed an analysis on the overall patient populations including both evaluable and unevaluable studies. For papers reporting only HR estimates obtained in multivariate analyses, we used this HR estimate together with its variance. For those with uncertainties related to the number of events and then the variance of the HR estimate, we made rough approximation of the variance. Finally, for the studies where no useful information could be retrieved from the publication, we considered that the HR estimate was 1 (i.e. no impact at all for Ki-67) and used a minimal variance compared to the included studies of the same size. Even by carrying out this sensitivity analysis, we still observe a significant pejorative impact of Ki-67 positivity on DFS (HR 1.74, 95% CI 1.56–1.95; *P*<0.001; heterogeneity test *P*<0.001) and OS (HR 1.76, 95% CI 1.54–2.00; *P*<0.001; heterogeneity test *P*<0.001).

## DISCUSSION

The present meta-analysis confirms that high Ki-67 expression in patients with early BC confers worse prognosis in the overall population and quantifies its prognostic univariate impact. Further, it was also shown in subgroup analyses for node-negative, node-positive and untreated patients. This is the first meta-analysis of published studies to evaluate the association between Ki-67/MIB-1 expression and prognosis in early BC. Prognostic markers may be defined as those markers that are associated with some clinical outcomes, typically a time-to-event outcome such as OS or DFS, independently of any treatment or intervention. The best setting to apply this concept is in untreated populations, which helps identifying the so-called pure prognostic marker. Prognostic markers may also be used to aid the decision-making process for adjuvant therapy, for example, they may be used as decision aids in determining whether a patient should receive adjuvant chemotherapy or how aggressive that therapy should be (McShane *et al*, 2005).

Ki-67 has been assayed in many studies as a prognostic and/or predictive marker in early BC. As a predictive marker, very few trials of primary systemic therapy, mostly retrospective and with conflicting results have been published (Colozza *et al*, 2005), and therefore we felt that the assessment of the predictive role of Ki-67 was out of scope for this meta-analysis.

Our meta-analysis was carried out using literature published results, and we therefore acknowledge some limitations of our approach which is, however, much less expensive than a meta-analysis using individual patients data. The language selection could favour positive studies, following the assumption that they are more often published in English, whereas the negative ones tend to be published more often in local journals using the author's native languages (Egger *et al*, 1997). However, we did not identify many papers published in a national language (Italian, Russian, Serbian, German) (Lelle, 1990; Topic *et al*, 2002; Kushlinskii *et al*, 2004; Costarelli *et al*, 2005). This may be called the ‘Tower of Babel bias’ and, in at least one of 36 consecutive meta-analyses, the exclusion of papers for linguistic reasons produced different results from those which would have been obtained if this exclusion criterion had not been used (Gregoire *et al*, 1995). Another possible source of confusion is the use of the same cohort of patients in different publications, although studies that were clearly based on the analysis of the same patient cohorts were excluded in this meta-analysis.

Some authors consider meta-analyses using individual data to be the gold standard evidence (Stewart and Parmar, 1993; Oxman *et al*, 1995). This approach is normally considered to be a new study that takes into account all performed studies on the topic, published or not, and that requires an individual data update by the investigators; it is thus much more time consuming, complex and costly. In a comparison between a meta-analysis based on individual patient data and one based on extracted data, the overall duration for the former was found to be 1–5 years while for the latter it is only 1–5 months. Additionally, the overall cost to perform an individual patient data meta-analysis is $50 000 to $500 000, whereas for an extracted data study it is in the range of $5000 to $30 000 (Piedbois and Buyse, 2004). Therefore, a meta-analysis on published literature is worthwhile and, especially in a situation, as here, it is very unlikely to find the resources to conduct a meta-analysis based on the individual data.

The method used for extrapolating HR might be a source of some variability in the HR estimates. When no other useful information was available, we extrapolated the HR from the survival curves using several time points during follow-up for reading the corresponding survival rates, assuming that censored observations were uniformly distributed. The estimation of survival rates based on the graphical representation of the survival curves was performed independently by three of the authors and we compared our HR estimate and its statistical significance with the results published in each individual trial. We did not identify any major contradiction between our results and the results available in the papers.

The adverse impact of Ki-67 positivity on both OS and DFS was observed in the overall population as well as in the subgroups node-negative and node-positive patients. Significant heterogeneity was detected when considering the whole population and node-negative patients. It is not considered appropriate to define a single measure (i.e. HR associated with Ki-67 positivity in this case) from studies with inherent dissimilarities. The observed disparity among the conclusions of different studies, responsible for the observed heterogeneity, can be quantified by applying quality scores to the selected studies included in the meta-analysis. However, these scores do not always explain the observed results (Greenland, 1994). In this case, the methodological characteristics of each study must be taken into consideration.

In 1992, Cattoretti *et al* (1992) reported better success in staining Ki-67 in paraffin-embedded samples after the new antibodies anti-MIB-1 and anti-MIB-3 had been developed. Although several antibodies are now commercially available to stain Ki-67, anti-MIB-1 is the most frequently used in recent studies (Urruticoechea *et al*, 2005). In our meta-analysis, antibodies other than anti-MIB-1 and anti-Ki-67 were included, such as anti-Ki-S5 (Rudolph *et al*, 1999a; Esteva *et al*, 2004) and anti-Ki-S11 (Rudolph *et al*, 1999b), albeit representing only a minority of the cases. Moreover, Ki-67 expression is usually estimated as the percentage of tumour cells positively stained by the antibody, with nuclear staining being the most common criteria of positivity. The use of different antibodies and scoring protocols without a standard minimum number of cells to be counted may account for some of the differences between the studies.

In our meta-analysis, some studies have used 10% as the cut-off (arbitrary value), whereas others have chosen mean, median, the optimal cut-off value or arbitrary values, and these differences might be responsible for the difficulty in determining a standard threshold in daily practice. However, some authors have described that the choice of the cut-off point for IHC may depend on the clinical objective: if Ki-67 is used to exclude patients with slowly proliferating tumours from chemotherapeutic protocols, a cut-off of 10% will help avoid overtreatment. In contrast, if Ki-67 is used to identify patients sensitive to chemotherapy protocols, it is preferable to set the cut-off at 25% (Spyratos *et al*, 2002). In the context of this meta-analysis, we may assume that increased Ki-67 leads to an increased risk of relapse and/or death and that a relative increase is estimated although the baseline risk (the risk in the group considered Ki-67 negative) is not the same in all the studies.

A further limitation of our meta-analysis is that it assesses only the univariate prognostic value of Ki-67. So, we cannot infer from our meta-analysis that Ki-67 is an independent factor; the answer to that question should come from a prospective study (it is likely that a meta-analysis of individual data would not solve the question as the intersection of the sets of covariates available in the individual studies is most probably very small).

To better clarify the prognostic role of ER status, Sotiriou *et al* (2006) used gene array profiling to explore the implications of the joint distribution of ER status and gene expression grade index (GGI) to predict clinical outcome. They found that almost all ER-negative tumours were associated with high GGI scores (high grade), whereas ER-positive tumours were associated with a heterogeneous mixture of GGI values. This means that GGI adds additional prognostic information when the ER status is known, whereas the opposite is not true. Unfortunately, due to the lack of information in the published studies used in our study, an analysis of the impact of Ki-67 expression on the ER-negative and ER-positive subpopulations and grade, which are well-known risk factor associated with worse outcome, was not possible. Table 5 summarises the main results of the recent genes signatures for prognosis/prediction in BC.

Despite years of research and hundreds of reports of tumour markers in oncology, the number of markers that have emerged as clinically useful is quite small. The REporting of tumour MARKer Studies (REMARK) guidelines was the major task of the NCI-EORTC First International Meeting on Cancer Diagnosis, representing a collaborative effort of statisticians, clinicians and laboratory scientists. The guidelines contain 20 recommendations derived from studies on tumour markers and regarding study design, methods of statistical analysis, preplanned hypotheses, patient and specimen characteristics, and assay methods. The widespread use of published guidelines for analytical methods and the reporting of results would greatly facilitate the development of alternative analyses and meta-analyses (Alonzo, 2005; McShane *et al*, 2005).

Despite some limitations, this meta-analysis supports the prognostic role of Ki-67 in early BC, by showing a significant association between its expression and the risk of recurrence and death in all populations considered and for both outcomes, DFS and OS. Had the proposed REMARK guidelines been employed in all the studies selected for this meta-analysis and had all necessary information been available, our literature-based meta-analysis would better characterise the role of Ki-67 as prognostic marker.

## Figures and Tables

**Table 1:** Main characteristics of all studies included in the meta-analysis for overall survival

**Author**	**Patients Ki-67+/− (total)**	**Median FU (mos)**	**Systemic treatment**	**Antibody**	**Threshold (chosen by)**	**HR (95% CI)**
Bevilacqua *et al* (1996)	94/13 (107)	74	Untreated	Anti-Ki-67 Anti-MIB-1	10% (arbitrary)	2.75 (1.02–7.39)
Bos *et al* (2003)	63/87 (150)	106 (mean)	N⩾4: CMF or TAM	Anti-Ki-67	10% (arbitrary)	2.47 (1.08–5.65)
Brown *et al* (1996)	170/504 (674)	72	156 CT and/or HT	Anti-Ki-67	5% (optimal cut-off)	1.19 (0.79–1.80)
Caly *et al* (2004)	122/122 (244)	72 (minimum)	Not reported	Anti-MIB-1	32% (proportion of scored cells)	1.95 (0.92–4.14)
Domagala *et al* (1996) N0	66/45 (111)	88	47 CT or HT	Anti-MIB-1	10% (median value)	3.04 (1.03–8.99)
Domagala *et al* (1996) N+	40/35 (75)	88	47 CT or HT	Anti-MIB-1	10% (median value)	1.38 (0.66–2.86)
Erdem *et al* (2005)	13/34 (47)	72.5	All adjuvant CT (?)	Anti-Ki-67	10% (median value)	17.23 (2.42–122.36)
Fresno *et al* (1997)	84/62 (146)	75	13 CMF 80 TAM	Anti-MIB-1	10% (arbitrary)	1.81 (0.71–4.59)
Gasparini *et al* (1994)	83/82 (165)	60	82 CMF and/or HT	Anti-Ki-67	7.5% (mean value)	2.58 (1.21–5.49)
Gonzalez *et al* (2003)	NR (221)	102.5	Not reported	Anti- MIB-1	30% (arbitrary)	3.18 (1.52–6.65)
Goodson *et al* (2000)	56/56 (112)	5.1 y	104 CT or HT	Anti- MIB-1	24% (mean value)	2.90 (1.18–7.15)
Heatley *et al* (2002)	26/33 (59)	5 y	Not reported	Anti-Ki-67	10% (mean value)	0.81 (0.36–1.81)
Hlupic *et al* (2004)	117/75 (192)	180 (for N+ patients)	Various adjuvant CT regimens (?)	Anti-Ki-67	10% (arbitrary)	1.30 (0.80–2.11)
Jacquemier *et al* (1998)	74/78 (152)	60	(?) FAC, FEC or FMC	Anti- MIB-1	3.5% (median value)	3.29 (1.49–7.22)
Jansen *et al* (1998)	153/168 (321)	128	(?) FAC	Anti-MIB-1	7% (median value)	1.35 (1.01–1.80)
Jensen *et al* (1995)	54/64 (118)	104	3 CT or HT	Anti-MIB-1	17% (median value)	3.41 (1.44–8.06)
Liu *et al* (2001)	389/384 (773)	16.3 y	268 CT (17% DOX)	Anti-MIB-1	17.8% (median value)	1.76 (1.41–2.20)
Locker *et al* (1992)	23/44 (67)	27	Not reported	Anti-Ki-67	9% (tertile distribution)	4.19 (1.19–14.7)
Mottolese *et al* (2000)	87/70 (157)	60	All EC	Anti-Ki-67	10% (arbitrary)	1.82 (0.90–3.67)
Pellikainen *et al* (2003)	184/230 (414)	57.2	(?) CMF and TAM or toremifene	Anti-MIB-1	20% (arbitrary)	2.56 (1.46–4.50)
Pierga *et al* (1996)	66/70 (136)	70	16 FAC/39 TAM	Anti-Ki-67	8% (median value)	1.37 (0.64–2.91)
Pietilainen *et al* (1996)	100/88 (188)	8.6 y (mean)	64 CT (?)	Anti-MIB-1	20% (arbitrary)	1.88 (1.16–3.05)
Pinder *et al* (1995)	74/103 (177)	NR	Untreated	Anti-MIB-1	34% (tertile distribution)	1.66 (1.09–2.52)
Pinto *et al* (2001)	136/159 (295)	39.6	201 CT/131 HT	Anti-Ki-67	10% (arbitrary)	1.46 (0.74–2.87)
Querzoli *et al* (1996)	43/127 (170)	66.5	Not reported	Anti-Ki-67 Anti-MIB-1	13% (tertile distribution)	2.05 (1.11–3.77)
Railo *et al* (1993)	37/289 (326)	2.7 y (mean)	Not reported	Anti-Ki-67	10% (nuclear staining)	2.39 (0.77–7.38)
Rudolph *et al* (1999a)	363/500 (863)	149.3	531 CT or HT	Anti-Ki-S11	25% (median values)	1.91 (1.50–2.44)
Rudolph *et al* (1999b)	137/234 (371)	95	86 TAM	Anti-Ki-S5	25% (median values)	4.88 (2.98–7.99)
Seshadri *et al* (1996)	235/472 (707)	66	(?) CMF or TAM	Anti-MIB-1	10% (arbitrary)	2.60 (1.80–3.75)
Thor *et al* (1999)	243/243 (486)	62	Not reported	Anti-MIB-1	28.6% (median value)	1.94 (1.04–3.61)
Trihia *et al* (2003) N0	61/127 (188)	13.5 y	125 CMF	Anti-MIB-1	16% (proportion of scored cells)	1.90 (1.18–3.08)
Trihia *et al* (2003) N+	82/164 (246)	13.5 y	246 CMF	Anti-MIB-1	16% (proportion of scored cells)	2.42 (1.71–3.41)
Tynninen *et al* (1999)	42/42 (84)	10.3 y (mean)	13 CT (?)	Anti-MIB-1	9.8% (median value)	1.05 (0.55–2.00)
Veronese *et al* (1995)	64/63 (127)	61	Not reported	Anti-Ki-67 Anti-MIB-1	14% (median value)	0.42 (0.20–0.87)
Weikel *et al* (1991)	78/115 (193)	23.6 (mean)	CMF and/or TAM	Anti-Ki-67	20% (proportion of scored cells)	3.42 (1.39–8.40)
Weikel *et al* (1995) N0	93/141 (234)	3.4 y (mean)	Mostly TAM	Anti-Ki-67	20% (groups)	1.66 (0.79–3.51)
Weikel *et al* (1995) N+	138/177 (315)	3.4 y (mean)	315 CMF and/or TAM	Anti-Ki-67	20% (groups)	2.36 (1.55–3.60)
Wintzer *et al* (1991)	32/31 (63)	37	Not reported	Anti-Ki-67	12% (median value)	2.51 (1.00–6.34)

CI, confidence interval; CMF, cyclophosphamide, methotrexate, 5-fluorouracil; CT, chemotherapy; DOX, doxorubicin; EC, epirubicin, cyclophosphamide; FAC, 5-fluorouracil, doxorubicin, cyclophosphamide; FEC, 5-fluorouracil, epirubicin, cyclophosphamide; FMC, 5-fluorouracil, mitoxantrone, cyclophosphamide; FU, follow-up; HR, hazard ratio; HT, hormonotherapy; mos, months; N0, node-negative; N+, node-positive; NR, not reported; TAM, tamoxifen; +, positive; −, negative; y, years.

**Table 2:** Main characteristics of all studies included in the meta-analysis for disease-free survival

**Author**	**Patients Ki-67± (total)**	**Median FU (mos)**	**Systemic Treatment**	**Antibody**	**Threshold (chosen by)**	**HR (95% CI)**
Bevilacqua *et al* (1996)	13/94 (107)	74	Untreated	Anti-Ki-67 Anti-MIB-1	10% (arbitrary)	3.95 (1.45–10.7)
Billgren *et al* (2002) N0	189/241(430)	5.7 y	149 CMF/484 TAM*	Anti-Ki-67 Anti-MIB-1	15% (arbitrary)	2.18 (1.04–4.57)
Billgren *et al* (2002) N+	168/134 (302)	5.7 y	149 CMF/484 TAM*	Anti-Ki-67 Anti-MIB-1	15% (arbitrary)	2.20 (1.28–3.78)
Bos *et al* (2003)	63/87 (150)	106 (mean)	*N*≥4: CMF or TAM	Anti-Ki-67	10% (arbitrary)	1.59 (0.83–3.04)
Bouzubar *et al* (1989)	65/59 (124)	3 (minimum)	Not reported	Anti-Ki-67	20% (arbitrary)	2.07 (0.99–4.30)
Brown *et al* (1996)	170/504 (674)	72	156 CT and/or HT	Anti-Ki-67	5% (optimal cut-off)	1.71 (1.18–2.47)
Caly *et al* (2004)	122/122 (244)	72 (minimum)	Not reported	Anti-MIB-1	32% (proportion of scored cells)	1.61 (1.01–2.55)
Clahsen *et al* (1999)	215/217 (430)	41	FAC (all)/ CMF for N+	Anti MIB-1	20% (arbitrary)	2.84 (1.80–4.47)
Erdem *et al* (2005)	13/34 (47)	72.5	All adjuvant CT (?)	Anti-Ki-67	10% (median value)	6.96 (2.62–18.44)
Esteva *et al* (2004)	29/61 (100)	11 y	FAC	Anti-Ki-S5	12% (proportion of scored cells)	1.42 (0.75–2.66)
Gaglia *et al* (1993) N0	90/90 (180)	31 (mean)	158 TAM	Anti-Ki-67	9% (median value)	4.60 (1.58–13.38)
Gaglia *et al* (1993) N+	87/86 (173)	31 (mean)	70 CMF /138 TAM	Anti-Ki-67	9% (median value)	1.87 (0.94–3.70)
Gasparini *et al* (1994)	83/82 (165)	60	82 CMF and/or HT	Anti-Ki-67	7.5% (median value)	3.21 (1.53–6.75)
Goodson *et al* (2000)	56/56 (112)	5.1 y	104 CT or HT	Anti-MIB-1	24% (mean value)	2.06 (0.95–4.45)
Harbeck *et al* (1999)	20/96 (116)	76	Untreated	Anti-Ki-67	25% (optimised values)	2.69 (1.09–6.62)
Jacquemier *et al* (1998)	74/78 (152)	60	(?) FAC, FEC or FMC	Anti-MIB-1	3.5% (median value)	2.81 (1.53–5.17)
Jansen *et al* (1998) N0	72/111 (183)	128	Untreated	Anti-MIB-1	7% (median value)	2.52 (1.50–4.22)
Jansen *et al* (1998) N+	81/57 (138)	128	(?) FAC	Anti-MIB-1	7% (median value)	1.34 (0.89–2.04)
Keshgegian and Cnaan (1995)	66/65 (131)	Up to 46 mos	Not reported	Anti-Ki-67 Anti-MIB-1	10% (arbitrary)	1.44 (0.50–4.10)
Lau *et al* (2001)	22/75 (97)	64 (mean)	Various adjuvant CT Regimens (?)	Anti-MIB-1	25% (arbitrary)	4.10 (1.33–12.55)
Liu *et al* (2001)	389/384 (773)	16.3 y	268 CT (17% DOX)	Anti-MIB-1	17.8% (median value)	1.69 (1.39–2.06)
Locker *et al* (1992)	23/44 (67)	27	Not reported	Anti-Ki-67	9% (tertile distribution)	2.04 (0.83–5.03)
Michalides *et al* (2002)	226/126 (352)	>8 y	Mostly TAM	Anti-MIB-1	5% (arbitrary)	2.06 (1.28–3.33)
Mottolese *et al* (2000)	87/70 (157)	60	All EC	Anti-Ki-67	10% (arbitrary)	1.52 (0.82–2.81)
Pellikainen *et al* (2003)	184/230 (414)	57.2	(?) CMF and TAM or toremifene	Anti-MIB-1	20% (arbitrary)	2.14 (1.36–3.38)
Pierga *et al* (1996) N0	30/48 (78)	70	Not reported	Anti-Ki-67	8% (median value)	1.89 (0.78–4.54)
Pierga *et al* (1996) N+	36/22 (58)	70	16 FAC/39 TAM	Anti-Ki-67	8% (median value)	0.95 (0.37–2.43)
Pietilainen *et al* (1996)	97/82 (179)	8.6 (mean)	64 CT (?)	Anti-MIB-1	20% (arbitrary)	1.60 (1.01–2.53)
Pinto *et al* (2001)	136/159 (295)	39.6	201 CT/131 HT	Anti-Ki-67	10% (arbitrary)	1.61 (0.93–2.80)
Querzoli *et al* (1996)	43/127 (170)	66.5	Not reported	Anti-Ki-67 Anti-MIB-1	13% (tertile distribution)	2.20 (1.25–3.87)
Railo *et al* (1993)	37/289 (326)	2.7 y (mean)	Not reported	Anti-Ki-67	10% (proportion of scored cells	3.38 (1.61–7.12)
Railo *et al* (1997)	89/123 (212)	8.3 y (mean)	Untreated	Anti-Ki-67	10% (arbitrary)	2.46 (1.33–4.56)
Rudolph *et al* (1999a)	363/500 (863)	149.3	531 CT or HT	Anti-Ki-S11	25% (median values)	1.98 (1.56–2.52)
Rudolph *et al* (1999b)	137/234 (371)	95	86 TAM	Anti-Ki-S5	25% (median values)	2.96 (1.92–4.57)
Sahin *et al* (1991)	14/28 (42)	88	Untreated	Anti-Ki-67	12% (3 groups)	4.54 (1.37–15.03)
Seshadri *et al* (1996)	235/472 (707)	66	(?) CMF or TAM	Anti-MIB-1	10% (arbitrary)	2.10 (1.50–2.93)
Thor *et al* (1999)	243/243 (486)	62	Not reported	Anti-MIB-1	28.6% (median value)	2.19 (1.45–3.30)
Trihia *et al* (2003) N0	61/127 (187)	13.5 y	125 CMF	Anti-MIB-1	16% (proportion of scored cells)	1.20 (0.78–1.84)
Trihia *et al* (2003) N+	82/164 (246)	13.5 y	246 CMF	Anti-MIB-1	16% (proportion of scored cells)	1.80 (1.31–2.47)
Veronese *et al* (1995)	64/63 (127)	61	Not reported	Anti-Ki-67 Anti-MIB-1	14% (median value)	0.60 (0.33–1.10)
Weikel *et al* (1991) N0	34/42 (76)	23.6 (mean)	Untreated	Anti-Ki-67	20% (proportion of scored cells)	1.75 (0.34–9.01)
Weikel *et al* (1991) N+	43/65 (108)	23.6 (mean)	CMF and/or TAM	Anti-Ki-67	20% (proportion of scored cells	0.71 (0.09–5.36)
Weikel *et al* (1995) N0	93/141 (234)	3.4 y (mean)	Mostly TAM	Anti-Ki-67	20% (groups)	1.10 (0.53–2.28)
Weikel *et al* (1995) N+	138/177 (315)	3.4 y (mean)	315 CMF and/or TAM	Anti-Ki-67	20% (groups)	1.51 (1.13–2.00)
Wintzer *et al* (1991)	32/31 (63)	37	Not reported	Anti-Ki-67	12% (median value)	2.99 (1.30–6.92)

CI, confidence interval; CMF, cyclophosphamide, methotrexate, 5-fluorouracil; CT, chemotherapy; DOX, doxorubicin; EC, epirubicin, cyclophosphamide; FAC, 5-fluorouracil, doxorubicin, cyclophosphamide; FEC, 5-fluorouracil, epirubicin, cyclophosphamide; FMC, 5-fluorouracil, mitoxantrone, cyclophosphamide; FU, follow-up; HR, hazard ratio; mos, months; N0, node-negative; N+, node-positive; NR, not reported; TAM, tamoxifen; y, years; +, positive; −, negative.

* For total population (*n*=732).

**Table 3:** HR values and heterogeneity test for all subgroups analysis in patients with early breast cancer

**Group**	**Number of studies**	**Number of patients**	**Fixed effect HR (95% CI)**	**Heterogeneity test (*P*-value)**	**Random effect HR (95% CI)**
Disease-free survival					
All pts	38	10 954	1.88 (1.75–2.02)	0.01	1.93 (1.74–2.14)
N− pts	15	3370	2.20 (1.88–2.58)	0.03	2.31 (1.83–2.92)
N+ pts	8	1430	1.59 (1.35–1.87)	0.68	
N− untreated pts	6	736	2.72 (1.97–3.75)	0.89	
					
*Overall survival*					
All pts	35	9472	1.89 (1.74–2.06)	<0.001	1.95 (1.70–2.24)
N− pts	9	1996	2.19 (1.76–2.72)	0.001	2.54 (1.65–3.91)
N+ pts	4	857	2.33 (1.83–2.95)	0.44	
N−/N+ untreated pts	2	284	1.79 (1.22–2.63)	0.36	

CI: confidence interval; HR: hazard ratio; N−: node-negative; N+: node-positive; pts: patients.

**Table 4:** Studies that were not evaluable for this meta-analysis, but included in the sensitivity test

**Author**	**Number of patients**	**Ki-67 prognostic value (Yes/No)**
Beck *et al* (1995)	462	Yes
Biesterfeld *et al* (1998)	103	Yes
Bukholm *et al* (2003)	147	No
Ceccarelli *et al* (2000)	217	Yes
Galiegue *et al* (2004)	117	No
Gasparini *et al* (1992)	164	Yes
Haerslev *et al* (1996)	487	Yes
Jalava *et al* (2000)	414	No
Kroger *et al* (2006)	157	No
Kronblad *et al* (2006)	377	Yes
Lampe *et al* (1998)	142	Yes
Liu *et al* (2000)	225	Yes
Michels *et al* (2003)	104	No
Molino *et al* (1997)	322	Yes
Rudas *et al* (1994)	184	No
Tsutsui *et al* (2005)	249	Yes
Yang *et al* (2003)	147	No

**Table 5:** Main results from the recent gene expression signatures in breast cancer

**Gene expression signature**	**Number of genes in the signature**	**Description of genes in the signature**	**Reference**
70-gene signature	70	Cell cycle, angiogenesis, invasion and metastasis	van't Veer *et al* (2002)
76-gene signature	76	Cell cycle, proliferation, DNA repair, immune response and apoptosis	Wang *et al* (2005)
Recurrence score	21	Proliferation, estrogen receptor and Her2 status, invasion and 5 reference genes	Paik *et al* (2004)
Genomic grade index	97	Cell cycle and proliferation genes	Sotiriou *et al* (2006)
p53-signature	32	Proliferation genes and transcription factors (not p53 targets)	Miller *et al* (2005)
Death from cancer signature	11	Cell cycle and proliferation genes	Glinsky *et al* (2005)
Estrogen-regulated gene expression signature	822	Proliferation and antiapoptosis genes	Oh *et al* (2006)
